# Risk factors of hepatitis C virus transmission and genotype distribution in former blood donors from Chinese rural area

**DOI:** 10.1186/s12889-015-1535-6

**Published:** 2015-02-25

**Authors:** Wenjiao Yin, Changhong Huang, Feng Qiu, Li Liu, Feng Wang, Jikun Zhou, Yong Zhang, Shengli Bi

**Affiliations:** Department of Viral Hepatitis, National Institute for Viral Disease Control and Prevention, Chinese Center for Disease Control and Prevention, Beijing, China; Kaifeng Center for Disease Control and Prevention, Kaifeng, Henan Province China; Shijiazhuang Center for Disease Control and Prevention, Shijiazhuang, Hebei Province China; Institute for Viral Disease Control and Prevention, Chinese Center for Disease Control and Prevention, Changping District, Beijing, 102206 PR China

**Keywords:** Hepatitis C virus, Blood donor, Risk factor, Phylogenetic tree

## Abstract

**Background:**

Illegal commercial plasma and blood donation activities in the late 1980s and early 1990s caused a large number of hepatitis C virus (HCV) infections in rural areas of China. In the present study, we aimed to elucidate the risk factors of HCV RNA positivity and HCV genotype distribution in former blood donors.

**Methods:**

A cross-sectional survey was carried out in a former blood donation village in rural Hebei Province, North China. All residents were invited for a questionnaire interview and testing for HCV antibodies as well as HCV nucleic acids. Questionnaires were administered to collect information about their personal status and commercial blood donation history. Nested PCR was used to amplify HCV nucleic acids in C/E1 region and NS5b region followed by genotyping and phylogenetic analysis. Univariate and multivariate logistic regression were used to analyze the distributions of HCV genotypes in different groups.

**Results:**

A total of 512 blood samples were collected. Anti-HCV positive were 148 (28.5%) whereas RNA positive rate was 13.87%. Residents between 50 and 59 years old had the highest RNA positive rate (27/109, 24.77%) (P = 0.0051). Multivariate logistic regression model analysis revealed that plasma donation (OR = 8.666, 95% CI: 1.390-54.025) was the dominant risk factor of HCV infection. Furthermore, HCV subtypes 1b and 2a were found by genotyping and phylogenetic analysis. 36 samples (53.73%) were subtype 1b and 31 samples (46.27%) were subtype 2a.

**Conclusions:**

Unsafe practices during illegal plasma donation led to a high risk of HCV infection. The identification of genotypes 1b and 2a as major HCV genotypes circulating in this region may help to predict the future burden of HCV related diseases and facilitate better medical treatment towards HCV carriers. These results are useful for public healthcare as well as disease control and surveillance.

**Electronic supplementary material:**

The online version of this article (doi:10.1186/s12889-015-1535-6) contains supplementary material, which is available to authorized users.

## Background

The hepatitis C virus (HCV) can cause hepatitis C, which can range in severity from a mild illness to lifelong diseases including liver cirrhosis or liver cancer [1]. About 130 to 170 million people are chronically infected with HCV worldwide [2,3]. In China, two nationwide sero-epidemiological studies were conducted. According to the latest investigation in 2006, the overall HCV prevalence in the population of 1–59 years old was 0.43% (95% CI: 0.33-0.53%), with about 4.29-6.89 million people estimated to be infected with HCV throughout China [4,5].

Hepatitis C virus is a single-stranded and positive-strand RNA virus. It is divided into six genotypes [6], which differ from each other by 31-33% at the nucleotide level. In addition, there are several subtypes with 20-25% differences [7]. The distribution of genotypes and subtypes varies geographically. For example, genotype 1b, 2a and 2b are widely distributed worldwide [8] whereas genotype 3 is mostly distributed in Indian subcontinent [9,10]. Genotype 4 is found in central Africa [11], genotype 5 is found in South Africa and genotype 6 is found in South Asia [12]. In China, studies have shown that genotypes 1b and 2a are the predominant HCV subtypes [13-15] and other subtypes (3a, 3b, 6a, 6n and 6u) were reported from southwest part of China [16,17].

Many residents in China rural areas were infected with HCV due to blood selling in the late 1980s. Although a previous study showed that the history of paid plasma donation was responsible for the high sero-prevalence of HCV in a village in rural Hebei Province, North China [18], little is known about the HCV RNA positivity and genotypes distribution in these blood donors. In the present study, we aimed to elucidate the risk factors for HCV RNA positivity and HCV genotypes distribution. The results may provide important information for future treatment of HCV related diseases and facilitate better public health control and surveillance.

## Methods

### Ethics statement

The survey protocol conformed to the ethical guidelines of the 1975 Declaration of Helsinki and was approved by the Ethics Committee in Chinese National Institute for Viral Disease Control and Prevention. The written informed consent of adults and permission from the parents of minors were obtained before the interview and venous blood collection.

### Questionnaire interview and serum sampling

The questionnaire included demographic characteristics of the subjects and exposure histories for possible risk factors related to HCV infection. Five milliliters of venous blood was then collected from each subject and the serum separated. Serum samples were stored at −20°C. A total of 520 residents were interviewed and 512 subjects accepted for venous blood collection. These subjects included former blood donors as well as non- blood donors, ranged in age from 3 to 89 years old; 261 were female (261/512, 50.98%) and 251 were male (251/512, 49.02%).

### Extraction and amplification of HCV RNA C/E1 region and NS5b region

HCV RNA was extracted from 140 μl serum with QIAamp Viral RNA Mini Kit (Qiagen, Hilden, Germany). HCV RNA positivity was identified by nested PCR based on C/E1 fragment. The PCR products were detected by 1% agarose gel electrophoresis for qualitative analysis. In addition, HCV genotypes and subgenotypes were determined by using nested PCR and sequencing. Briefly, two fragments corresponding to the HCV genome sequence nt 845–1315 on H77 (fragment C/E1) and nt 8254–8636 (fragment NS5b), were amplified respectively. A typical amplification procedure was performed in a 50 μl reaction volume containing 5 μl extracted HCV RNA and *Taq* polymerase for 35 cycles, denaturing for 30 s at 94°C, annealing for 30 s at 58°C, and elongation for 35 s at 72°C, followed by a final extension at 72°C for 10 min. Standard precautions to avoid contamination during PCR were taken, including a negative control serum in each run. The primers used for the amplification of fragment C/E1 and fragment NS5b was listed in Table 1.

**Table 1:** **Details of the primers for HCV amplification**

**Targeted fragment**	**Name**	**Sequence of oligonucleotide (5’-3’)**	**Position (on H77)**
Fragment C/E1	C/E1-F1	ACTGCCTGATAGGGTGCTTGC	288-308
	C/E1-R1	GGTGACCAGTTCATCATCATATCC	1301-1324
	C/E1-F2	CCTTCCTGGTTGCTCTTTCTCTAT	845-868
	C/E1-R2	GTTCATCATCATATCCCAAGCCAT	1293-1315
Fragment NS5b	NS5b-F1	CCAATTVVCACTACHATCATGGC	7995-8017
	NS5b-R1	TGGRGTGTGTCKDGCTGTTTCC	8789-8810
	NS5b-F2	CGTATGATACCCGCTGTTTTGA	8254-8275
	NS5b-R2	CCTGGTCATAGCCTCCGTGAA	8616-8636

### Nucleotide sequencing of PCR products

The positive amplification products identified by agarose gel electrophoresis were sequenced directly by the dideoxy termination method using the Big Dye Terminator cycle sequencing Ready Reaction Kit ver. 3.1 (Applied Biosystems, Foster City, CA) with modifications and an automatic sequencer (ABI PRISM 3100 Genetic Analyser; Applied Biosystems). PCR products were sequenced in forward and reverse directions. Sequence alignment was performed by using the multiple-alignment algorithm in MegAlign software (DNASTAR, Windows version 5.06, WI).

### HCV genotyping and phylogenetic analysis

The PCR product of fragment C/E1 was 471 bp and that of fragment NS5b was 383 bp. The nucleotide sequences obtained were aligned with HCV strains of standard genotypes by the ClustalW method of MEGA software (Version 4.0), and the Neighbor-Joining method was used to construct a phylogenetic tree to determine the genotypes of our samples.

### Statistical analysis

Univariate and multivariate logistic regression were used to analyze associations between HCV RNA positivity and risk factors. Statistical analysis was performed using SAS 9.1 (Statistical Analysis Software Institute Inc, USA).

## Results

### Risk factor analysis of HCV RNA positivity in former blood donors

HCV RNA positivity was found in 71 (71/512, 13.87%) of the participants, with 37 (37/261, 14.18%) females and 34 (34/251, 13.55%) males. There was no statistical difference between male and female (P = 0.8365). However, there were significant differences between age groups (P = 0.0051). The prevalence of HCV RNA positivity was 2.08% in 3–19 years age group, 9.52% in 20–29 years age group, 6.78% in 30–39 years age group, 14.06% in 40–49 years age group, 24.77% in 50–59 years age group and 14.29% in >60 years age group. Furthermore, there was a statistically higher prevalence in married people (68/449, 15.14%), compared to unmarried people (3/63, 4.76%) (P = 0.0358). There was no significant difference by occupation (P = 0.094), as the prevalence was 16.88% in peasants, 10.17% in students, 6.25% in preschool children, and 8.55% in people whose occupation were unknown. There was also no statistical difference by education degree (P = 0.2540), as the prevalence was 19.80% in illiterate people, 15.60% in primary school level, 13.04% in junior high school level, 15.63% in senior high school level, 6.67% in kindergarten level, and 8.55% in people whose education level was unknown (Table 2).

**Table 2:** **Univariate analysis of HCV RNA positivity and related factors**

**Variable**	**No. of samples**	**No. of positive**	**Positive rate (%)**	**OR (95% CI)**	***P*** **value**
**Sex**					0.8365
Female	261	37	14.18	Reference	
Male	251	34	13.55	0.949(0.574,1.567)	
**Age group**					0.0051
3-19 years	48	1	2.08	Reference	
20-29 years	63	6	9.52	4.947(0.575,42.555)	
30-39 years	59	4	6.78	3.418(0.369,31.650)	
40-49 years	128	18	14.06	7.691(0.998,59.292)	
50-59 years	109	27	24.77	15.476(2.037,117.583)	
60 years or older	105	15	14.29	7.833(1.004,61.138)	
**Marriage status**					0.0358
unmarried	63	3	4.76	Reference	
married	449	68	15.14	3.570(1.088,11.709)	
**Occupation**					0.0940
Peasant	320	54	16.88	Reference	
Student	59	6	10.17	0.558(0.228,1.363)	
Preschool child	16	1	6.25	0.328(0.042,2.539)	
Unknown	117	10	8.55	0.460(0.226,0.937)	
**Education degree**					0.2540
Illiterate	101	20	19.80	Reference	
Primary school	109	17	15.60	0.748(0.367,1.526)	
Junior high school	138	18	13.04	0.607(0.303,1.219)	
Senior high school	32	5	15.63	0.750(0.257,2.192)	
Kindergarten	15	1	6.67	0.289(0.036,2.332)	
Unknown	117	10	8.55	0.379(0.168,0.853)	
**Whole blood donation**					0.0102
No	399	64	16.04	Reference	
Yes	113	7	6.19	0.346(0.154,0.777)	
**Plasma donation**					0.1177
No	507	69	13.61	Reference	
Yes	5	2	40.00	4.232(0.695,25.785)	
Mix donation*					<0.0001
No	398	30	7.54	Reference	
Yes	114	41	35.96	6.889(4.040,11.748)	
**Blood transfusion**					0.6913
No	507	70	13.81	Reference	
Yes	5	1	20.00	1.563(0.172,14.175)	
**Tooth-brush share**					0.5269
No	508	70	13.78	Reference	
Yes	4	1	25.00	2.086(0.214,20.334)	
**Razor share**					0.9739
No	505	70	13.86	Reference	
Yes	7	1	14.29	1.036(0.123,8.734)	
**Therapeutic injection**					0.5103
No	478	65	13.60	Reference	
Yes	34	6	17.65	1.362(0.543,3.416)	
**Acupuncture**					0.3609
No	493	67	13.59	Reference	
Yes	19	4	21.05	1.696(0.546,5.263)	
**Surgery operation**					0.3566
No	435	58	13.33	Reference	
Yes	77	13	16.88	1.363(0.706,2.634)	
**Tooth extraction**					0.8907
No	469	65	13.86	Reference	
Yes	43	6	13.95	1.065(0.431,2.633)	

*Note: Mix donation denotes whole blood donation and plasma donation.

In regard to risk behavior variables and HCV infection, it was important to note that the selling of blood was prominent in this district in the late 1980s. Two types of blood selling were found in this village, the selling of whole blood and the selling of plasma. The results of our questionnaire indicated 232 people had a history of selling whole blood or plasma, with 50 serum samples (50/232, 21.55%) positive for HCV RNA. According to the types of blood selling, HCV RNA positivity was detected in 7 subjects (7/113, 6.19%) who had sold whole blood, 2 subjects (2/5, 40.00%) who had sold plasma, 41 subjects (41/114, 35.96%) who had sold whole blood and plasma, and 21 subjects (21/280, 7.5%) who had neither sold blood nor plasma. In contrast, histories of blood transfusion, toothbrush sharing, razor sharing, therapeutic injection, acupuncture, surgical operations, or tooth extraction were not risk indicators for HCV RNA positivity in our analysis (P > 0.05) (Table 2).

From a multivariate logistic regression model of HCV RNA positivity, histories of plasma donation (OR = 8.666, 95% CI: 1.390-54.025) and mixed donation (OR = 7.402, 95% CI: 4.301-12.740) were risk factors for HCV RNA positivity (Table 3).

**Table 3:** **Logistic regression of multi-risk factors for HCV RNA positivity**

**Variable**	**OR (95% CI)**	***P*** **value**
**Plasma donation**		0.0207
No	Reference	
Yes	8.666(1.390,54.025)	
Mix donation*		<0.0001
No	Reference	
Yes	7.402(4.301,12.740)	

*Note: Mix donation denotes whole blood donation and plasma donation.

### HCV genotype distribution and phylogenetic analysis

**Figure 1:**
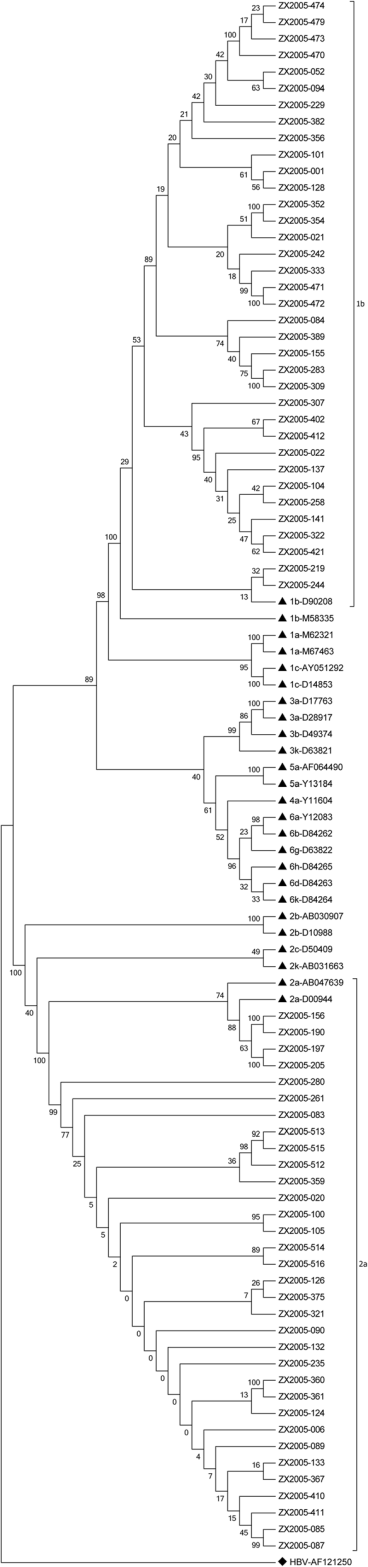
**Phylogenetic tree of HCV C/E1 sequences.**

**Figure 2:**
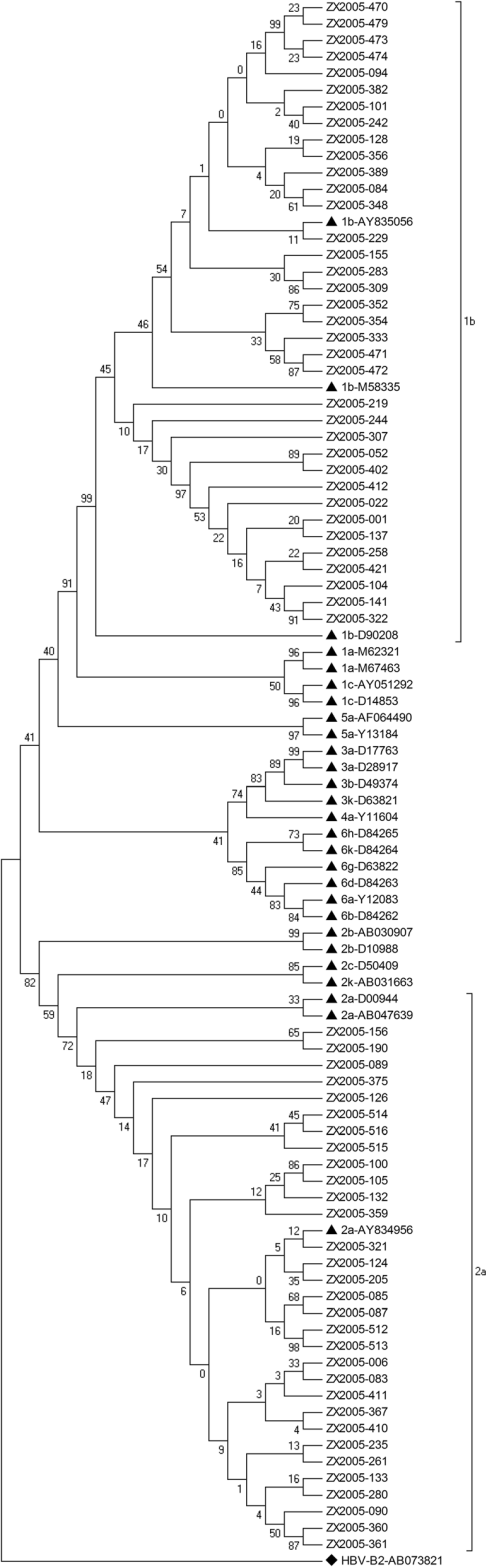
**Phylogenetic tree of HCV NS5b sequences.**

Sequencing based on C/E1 region and NS5b region of HCV genome and the phylogenetic analysis were performed successfully for 67 HCV RNA-positive samples. The results demonstrated that 36 strains (36/67, 53.73%) were closely related to subtype 1b, and 31 strains (31/67, 46.27%) belonged to subtype 2a. In genotype 1b isolates, ZX2005-229 was highly homologous with reference strain AY835056 which was isolated in Kunming in 2005. Comparatively, ZX2005-321 in 2a isolates was strongly consistent with AY834956 which was a Beijing HCV strain with 99% homology. The mean pairwise distance of these 67 strains was 0.544 substitutions per nucleotide site, with 0.060 in genotype 1b strains and 0.030 in genotype 2a strains, respectively (Figures 1 and 2).

The phylogenetic tree based on HCV C/E1 sequences and NS5b sequences demonstrated that 36 strains were closely related to subtype 1b and 31 strains belonged to subtype 2a. ▲ denotes the reference isolates and the bootstrap values were added. ♦ denotes that a HBV strain (accession number: AF121250 & AB073821) was used as an out-group.

Furthermore, all HCV isolates discovered in this study were uploaded to NCBI website and the Accession Numbers released by NCBI were: KP721870 to KP721938 for sequences from the amplification of C/E1 region and KP721803 to KP721869 for sequences of NS5b region respectively (Additional file 1).

By using NCBI BLAST, it was indicated that for genotype 1b strains, 16 strains (16/36, 44.44%) were closely related to isolate km48 (accession number: AY835056), with 97-99% homology, 3 strains were related to isolate HC-P32(accession number: AF046186), 3 strains were related to isolate HC-C2(accession number: D10934), and the remaining 14 strains were related to other isolates. For genotype 2a strains, 27 strains (27/31, 87.10%) were closely related to isolate bj779 (accession number: AY834956), with 96-99% homology, 4 strains (4/31, 12.90%) were closely related to isolate ZS199 (accession number: JX677261), GZ302 (accession number: JX521943), ZS37 (accession number: JX677354) or P012-NL(AE)_2a_1977.7 (accession number: JF722462).

Further analysis indicated HCV genotype distribution in gender and age groups. Briefly, in females, 14 strains (14/34, 41.18%) belonged to genotype 1b, and 20 strains (20/34, 58.82%) belonged to genotype 2a; however, 22 strains (22/33, 66.67%) belonged to genotype 1b, and 11 strains (11/33, 33.33%) belonged to genotype 2a in males. The distribution of HCV genotypes statistically differed by gender (χ^2^ = 4.3767, P = 0.0364), but did not significantly differ by age (Fisher’s Exact Test, P = 0.7987).

## Discussion

The total HCV RNA positive rate was much higher in the village examined compared to the general Chinese population [4,5] due to illegal blood donation that prevailed in some areas of Hebei province in the late 1980s [19,20]. According to results from a logistic regression model for HCV RNA positivity, illegal blood donation, especially plasma donation, probably led to the high positive rate of HCV RNA. Plasma donation is a blood collection method in which plasma is separated by centrifugation and the remaining cells transfused back into donors. This process could easily cause cross-infection due to the poor disinfection of blood collectors and substandard aseptic technology. Importantly, the HCV RNA positive rate of plasma donors was 40% and the mixed donors who sold whole blood and plasma had a rate of 35.96%. In the middle 1990s, the Chinese government enacted laws to ban this type of blood donation. Although illegal blood collection agencies in this region were banned, the prevalence of HCV RNA was still high because of the chronic infection of HCV. Males and females had an equal positive rate of HCV RNA, which could reflect their common behavior in selling blood. Males and females typically sold blood together and thus had an equal risk of being infected by blood-borne viruses.

Classified by age, residents 50–59 years old had the highest positive rate of HCV RNA, whereas the 3–19 year old population had the lowest. Blood donors in the late 1980s were mostly young adults responsible for supporting families; therefore, they usually sold their blood because of their poverty and low literacy.

HCV subtype 1b was predominant in this village and the next prevalent genotype was 2a. Other subtypes were not found in this study. Previous studies showed that genotypes 1b and 2a are dominant in China, and some studies reported the presence of genotypes 6a and 3b in southern China [13-15]. The village in this study was located in Hebei Province in northern China. In the late 1980s, many illegal blood agencies set up blood collection sites in this village and many local peasants went there to sell blood. We speculate that HCV subtype 1b and 2a were transmitted in this village and nearby regions. Cross infection during the blood drawing process likely caused the transmission of different HCV subtypes. Reports have indicated that patients with HCV genotype 1a or 1b had more severe liver disease and lower response rates to interferon therapy than patients with HCV genotype 2a or 2b [17]. Compared to genotype 1b, genotype 2a may be easier to clear spontaneously or by antiviral treatment. This may partly explain why the proportion of genotype 1b was slightly higher than genotype 2a.

Interestingly, HCV subtype 2a was predominant in females and subtype 1b was predominant in males. The cause of this kind of distribution is unknown, but there are two possibilities, firstly, only HCV RNA positive samples were genotyped and may have limited sample size. Secondly, females may be more susceptible to subtype 2a and males more vulnerable to subtype 1b though this latter hypothesis is highly speculative.

More than 40% of subtype 1b samples were closely related to isolate km48, and 3 strains were closely related to isolate HC-C2 [21]. Similar to previous studies [13-15], we speculate that subtype 1b strains in our study may be widely distributed in China. Compared to subtype 2a strains in our study, the mean pairwise distance was higher, which indicated the genetic diversity of subtype 1b samples in this village.

We believe most of our 2a strains may have a common origin with isolate bj779 which was isolated from a subject in Beijing because about 87.10% of the subtype 2a strains were closely related to isolate bj779, with a mean pairwise distance of only 0.030. We also speculate that subtype 2a strains similar to the isolates in our study may be widely distributed in China [22].

## Conclusion

In conclusion, unsafe practices during illegal plasma donation led to a high risk of HCV infection. The present epidemiological investigation may help to predict the future burden of HCV related diseases and facilitate better public health control and surveillance. Presently, those residents positive for HCV RNA need urgent medical assistance. The identification of genotypes 1b and 2a as major HCV genotypes circulating in this region represents valuable information for future medical treatment. It is desirable that China government may provide financial support and free healthcare to these villages, because most of the residents in this rural district make their living by farming and have no stable economic resources.

## Additional file

Additional file 1:
**NCBI accession numbers of the HCV C/E1 and NS5b sequences isolated in this study.**

## Abbreviations

HCV: Hepatitis C virus
PCR: Polymerase chain reaction
