# Supplementary material for: Risk factors of hepatitis C virus transmission and genotype distribution in former blood donors from Chinese rural area
Source: BMC Public Health. 2015 Feb 25;15:184. doi: 10.1186/s12889-015-1535-6 (PMC4355568; doi:10.1186/s12889-015-1535-6)
Supplement: Additional file 1: — NCBI accession numbers of the HCV C/E1 and NS5b sequences isolated in this study. [file 12889_2015_1535_MOESM1_ESM.doc]

NS5B.sqn ZX2005-001 KP721803 CE1.sqn ZX2005-001 KP721870

NS5B.sqn ZX2005-006 KP721804 CE1.sqn ZX2005-006 KP721871

CE1.sqn ZX2005-020 KP721872

CE1.sqn ZX2005-021 KP721873

NS5B.sqn ZX2005-022 KP721805 CE1.sqn ZX2005-022 KP721874

NS5B.sqn ZX2005-052 KP721806 CE1.sqn ZX2005-052 KP721875

NS5B.sqn ZX2005-083 KP721807 CE1.sqn ZX2005-083 KP721876

NS5B.sqn ZX2005-084 KP721808 CE1.sqn ZX2005-084 KP721877

NS5B.sqn ZX2005-085 KP721809 CE1.sqn ZX2005-085 KP721878

NS5B.sqn ZX2005-087 KP721810 CE1.sqn ZX2005-087 KP721879

NS5B.sqn ZX2005-089 KP721811 CE1.sqn ZX2005-089 KP721880

NS5B.sqn ZX2005-090 KP721812 CE1.sqn ZX2005-090 KP721881

NS5B.sqn ZX2005-094 KP721813 CE1.sqn ZX2005-094 KP721882

NS5B.sqn ZX2005-100 KP721814 CE1.sqn ZX2005-100 KP721883

NS5B.sqn ZX2005-101 KP721815 CE1.sqn ZX2005-101 KP721884

NS5B.sqn ZX2005-104 KP721816 CE1.sqn ZX2005-104 KP721885

NS5B.sqn ZX2005-105 KP721817 CE1.sqn ZX2005-105 KP721886

NS5B.sqn ZX2005-124 KP721818 CE1.sqn ZX2005-124 KP721887

NS5B.sqn ZX2005-126 KP721819 CE1.sqn ZX2005-126 KP721888

NS5B.sqn ZX2005-128 KP721820 CE1.sqn ZX2005-128 KP721889

NS5B.sqn ZX2005-132 KP721821 CE1.sqn ZX2005-132 KP721890

NS5B.sqn ZX2005-133 KP721822 CE1.sqn ZX2005-133 KP721891

NS5B.sqn ZX2005-137 KP721823 CE1.sqn ZX2005-137 KP721892

NS5B.sqn ZX2005-141 KP721824 CE1.sqn ZX2005-141 KP721893

NS5B.sqn ZX2005-155 KP721825 CE1.sqn ZX2005-155 KP721894

NS5B.sqn ZX2005-156 KP721826 CE1.sqn ZX2005-156 KP721895

NS5B.sqn ZX2005-190 KP721827 CE1.sqn ZX2005-190 KP721896

CE1.sqn ZX2005-197 KP721897

NS5B.sqn ZX2005-205 KP721828 CE1.sqn ZX2005-205 KP721898

NS5B.sqn ZX2005-219 KP721829 CE1.sqn ZX2005-219 KP721899

NS5B.sqn ZX2005-229 KP721830 CE1.sqn ZX2005-229 KP721900

NS5B.sqn ZX2005-235 KP721831 CE1.sqn ZX2005-235 KP721901

NS5B.sqn ZX2005-242 KP721832 CE1.sqn ZX2005-242 KP721902

NS5B.sqn ZX2005-244 KP721833 CE1.sqn ZX2005-244 KP721903

NS5B.sqn ZX2005-258 KP721834 CE1.sqn ZX2005-258 KP721904

NS5B.sqn ZX2005-261 KP721835 CE1.sqn ZX2005-261 KP721905

NS5B.sqn ZX2005-280 KP721836 CE1.sqn ZX2005-280 KP721906

NS5B.sqn ZX2005-283 KP721837 CE1.sqn ZX2005-283 KP721907

NS5B.sqn ZX2005-307 KP721838 CE1.sqn ZX2005-307 KP721908

NS5B.sqn ZX2005-309 KP721839 CE1.sqn ZX2005-309 KP721909

NS5B.sqn ZX2005-321 KP721840 CE1.sqn ZX2005-321 KP721910

NS5B.sqn ZX2005-322 KP721841 CE1.sqn ZX2005-322 KP721911

NS5B.sqn ZX2005-333 KP721842 CE1.sqn ZX2005-333 KP721912

NS5B.sqn ZX2005-348 KP721843

NS5B.sqn ZX2005-352 KP721844 CE1.sqn ZX2005-352 KP721913

NS5B.sqn ZX2005-354 KP721845 CE1.sqn ZX2005-354 KP721914

NS5B.sqn ZX2005-356 KP721846 CE1.sqn ZX2005-356 KP721915

NS5B.sqn ZX2005-359 KP721847 CE1.sqn ZX2005-359 KP721916

NS5B.sqn ZX2005-360 KP721848 CE1.sqn ZX2005-360 KP721917

NS5B.sqn ZX2005-361 KP721849 CE1.sqn ZX2005-361 KP721918

NS5B.sqn ZX2005-367 KP721850 CE1.sqn ZX2005-367 KP721919

NS5B.sqn ZX2005-375 KP721851 CE1.sqn ZX2005-375 KP721920

NS5B.sqn ZX2005-382 KP721852 CE1.sqn ZX2005-382 KP721921

NS5B.sqn ZX2005-389 KP721853 CE1.sqn ZX2005-389 KP721922

NS5B.sqn ZX2005-402 KP721854 CE1.sqn ZX2005-402 KP721923

NS5B.sqn ZX2005-410 KP721855 CE1.sqn ZX2005-410 KP721924

NS5B.sqn ZX2005-411 KP721856 CE1.sqn ZX2005-411 KP721925

NS5B.sqn ZX2005-412 KP721857 CE1.sqn ZX2005-412 KP721926

NS5B.sqn ZX2005-421 KP721858 CE1.sqn ZX2005-421 KP721927

NS5B.sqn ZX2005-470 KP721859 CE1.sqn ZX2005-470 KP721928

NS5B.sqn ZX2005-471 KP721860 CE1.sqn ZX2005-471 KP721929

NS5B.sqn ZX2005-472 KP721861 CE1.sqn ZX2005-472 KP721930

NS5B.sqn ZX2005-473 KP721862 CE1.sqn ZX2005-473 KP721931

NS5B.sqn ZX2005-474 KP721863 CE1.sqn ZX2005-474 KP721932

NS5B.sqn ZX2005-479 KP721864 CE1.sqn ZX2005-479 KP721933

NS5B.sqn ZX2005-512 KP721865 CE1.sqn ZX2005-512 KP721934

NS5B.sqn ZX2005-513 KP721866 CE1.sqn ZX2005-513 KP721935

NS5B.sqn ZX2005-514 KP721867 CE1.sqn ZX2005-514 KP721936

NS5B.sqn ZX2005-515 KP721868 CE1.sqn ZX2005-515 KP721937

NS5B.sqn ZX2005-516 KP721869 CE1.sqn ZX2005-516 KP721938
